# Whole-Body EMS Superimposed Walking and Nordic Walking on a Treadmill—Determination of Exercise Intensity to Conventional Exercise

**DOI:** 10.3389/fphys.2021.715417

**Published:** 2021-10-04

**Authors:** Ronald Verch, Josephine Stoll, Miralem Hadzic, Andrew Quarmby, Heinz Völler

**Affiliations:** ^1^Clinical Exercise Science, University Outpatient Clinic Potsdam, Department Sports and Health Sciences, University of Potsdam, Potsdam, Germany; ^2^University Outpatient Clinic, Sports Medicine and Sports Orthopaedics, Department Sports and Health Sciences, University of Potsdam, Potsdam, Germany; ^3^Department of Rehabilitation Medicine, Faculty of Health Science Brandenburg, University of Potsdam, Potsdam, Germany; ^4^Department of Cardiology, Klinik am See, Rüdersdorf, Germany

**Keywords:** electrical muscle stimulation, walking, Nordic walking, treadmill, exercise intensity

## Abstract

Electrical muscle stimulation (EMS) is an increasingly popular training method and has become the focus of research in recent years. New EMS devices offer a wide range of mobile applications for whole-body EMS (WB-EMS) training, e.g., the intensification of dynamic low-intensity endurance exercises through WB-EMS. The present study aimed to determine the differences in exercise intensity between WB-EMS-superimposed and conventional walking (EMS-CW), and CON and WB-EMS-superimposed Nordic walking (WB-EMS-NW) during a treadmill test. Eleven participants (52.0 ± years; 85.9 ± 7.4 kg, 182 ± 6 cm, BMI 25.9 ± 2.2 kg/m^2^) performed a 10 min treadmill test at a given velocity (6.5 km/h) in four different test situations, walking (W) and Nordic walking (NW) in both conventional and WB-EMS superimposed. Oxygen uptake in absolute (VO_2_) and relative to body weight (rel. VO_2_), lactate, and the rate of perceived exertion (RPE) were measured before and after the test. WB-EMS intensity was adjusted individually according to the feedback of the participant. The descriptive statistics were given in mean ± SD. For the statistical analyses, one-factorial ANOVA for repeated measures and two-factorial ANOVA [factors include EMS, W/NW, and factor combination (EMS^*^W/NW)] were performed (α = 0.05). Significant effects were found for EMS and W/NW factors for the outcome variables VO_2_ (EMS: *p* = 0.006, *r* = 0.736; W/NW: *p* < 0.001, *r* = 0.870), relative VO_2_ (EMS: *p* < 0.001, *r* = 0.850; W/NW: *p* < 0.001, *r* = 0.937), and lactate (EMS: *p* = 0.003, *r* = 0.771; w/NW: *p* = 0.003, *r* = 0.764) and both the factors produced higher results. However, the difference in VO_2_ and relative VO_2_ is within the range of biological variability of ± 12%. The factor combination EMS^*^W/NW is statistically non-significant for all three variables. WB-EMS resulted in the higher RPE values (*p* = 0.035, *r* = 0.613), RPE differences for W/NW and EMS^*^W/NW were not significant. The current study results indicate that WB-EMS influences the parameters of exercise intensity. The impact on exercise intensity and the clinical relevance of WB-EMS-superimposed walking (WB-EMS-W) exercise is questionable because of the marginal differences in the outcome variables.

## Introduction

Electrical muscle stimulation (EMS) is an increasingly popular training method and has become the focus of research in recent years. EMS is the electrical excitation and contraction of a muscle or a muscle group through an external applied electric field (Teschler and Mooren, 2019). Compared to voluntary exercise, which represents a progressive muscle fiber recruitment from small to large muscles according to the intensity of considered contraction, EMS represents a non-selective recruitment pattern of primarily fast-twitch motor units (muscle fibers type 2) at relatively low force levels (Henneman et al., 1965; Gregory and Bickel, 2005). Therefore, greater strength and power adaptation by synchronous recruitment of muscle fibers and a higher firing rate is possible (Gregory and Bickel, 2005). Otherwise, it is assumed that the application of EMS is just beneficial for submaximal and not for maximal exercise intensities (Paillard, 2018).

Electrical muscle stimulation devices are in a constant state of technological development. Compared to the devices used in previous studies (Buuren et al., 2013, 2015; Kemmler et al., 2013, 2016b,c), which require a connection between the main station, vest, and belts worn by the patient for power transfer *via* cable, newer EMS-devices work wirelessly *via* battery and apps for intensity adjusting. These new EMS devices offer a wide range of mobile applications for whole-body EMS (WB-EMS) training. Therefore, the use of superimposed WB-EMS for intensification of dynamic exercises has become more prominent in research (Wahl et al., 2014) with contradictory results. The previous studies have shown that superimposed WB-EMS can intensify voluntary bodyweight resistance training (Watanabe et al., 2019) and lead to the higher metabolic and respiratory response in its application during ergometer cycling (Mathes et al., 2017) and increased metabolic stress and hormonal responses (Wahl et al., 2015). In contrast, beneficial effects regarding the strength and power parameters are debatable. Superimposed dynamic WB-EMS resistance training seems to provide minor or no benefits when compared with dynamic resistance training alone (Micke et al., 2018).

Apart from the changing of dietary habits, endurance training is an important part of the management of several diseases and metabolic disorders such as type 2 diabetes mellitus (T2DM) (Hopps et al., 2011; Beavers et al., 2013). However, different reasons could lead to the physical inactivity of DMT2 patients. Previous studies identified walking as the most popular and most preferred exercise among these patients (Ford and Herman, 1995; Thomas et al., 2004). As a low-intensity exercise, it has various advantages compared with running and cycling. However, only minor beneficial metabolic effects for this patient group have been shown, indicating that the exercise intensity of normal walking might be insufficient (Karstoft et al., 2013). Furthermore, Nordic walking (NW) represents another approach for exercise in T2DM patients. At a defined speed, Nordic walking involves more muscles in various body segments and induces greater exercise intensity in terms of higher absolute oxygen uptake (VO_2_) compared with level walking (Sugiyama et al., 2013). Different reviews examining the health benefits of NW have confirmed these results. NW exerts beneficial effects on resting heart rate, blood pressure, exercise capacity, maximal oxygen consumption, and quality of life in patients with various diseases and can thus be recommended to a wide range of people as primary and secondary prevention (Tschentscher et al., 2013; Mathieson and Lin, 2014).

However, as already shown in other dynamic exercises, the question arises whether superimposed WB-EMS has also the potential to intensify W and NW resulting in higher metabolic response with potential therapeutic application in different patient cohorts. Therefore, the present study aimed to determine the differences in exercise intensity between WB-EMS-superimposed and conventional walking (WB-EMS-CON-W) and CON and WB-EMS-superimposed Nordic walking (WB-EMS-NW) during a treadmill test with a constant given velocity in a cohort of healthy men. We assume that WB-EMS-superimposed walking and Nordic walking (WB-EMS-W/NW) result in higher VO_2_ compared with the CON exercise execution. We also assume that due to the higher recruitment in various body segments during NW, WB-EMS has a higher impact on NW compared to W.

## Materials and Methods

### Study Design

The study was conducted as a randomized crossover design with a total of four measurement appointments. To avoid WB-EMS-induced delayed onset muscle soreness (DOMS) as a confounding factor on test performance (Nosaka et al., 2002; Mackey et al., 2008; Vanderthommen et al., 2012; Kemmler et al., 2015), an interval of 1 week between each of the four measurement appointments (M1–M4) was chosen. During the four measurements, the four test situations (CON-W, EMS- W, CON-NW, and EMS-NW) were conducted in random order. Randomization was carried out with randomization software (www.randomizer.org).

After participants were informed about the study background, aims, procedure, risks of the participation, and signing the written informed consent, every participant had a medical entrance examination during the first appointment (M1) to ensure their ability to perform the trial. During this examination, body weight (BW) and height were measured, and a physician performed a basic orthopedic and cardiopulmonary checkup which included a resting electrocardiogram. Blood samples were also taken to exclude acute infection.

### Subjects

The recruitment of participants took place in the environment of the research facility and a local sports center. Healthy, non-smoking men between the age of 35 and 70 years with a moderate activity profile (<3 h/week) were included. Regular drug consumption, cardiac arrhythmias (Lown classification ≥ 3), heart failure (New York Heart Association classification ≥ II), and wearing an implantable cardiac device were exclusion criteria. Additional exclusion criteria were diseases, such as chronic obstructive pulmonary disease (COPD), movement-restricting diseases, and rheumatic and neurological diseases. Eleven healthy men (52.0 ± years; 85.9 ± 7.4 kg, 182 ± 6 cm, BMI 25.9 ± 2.2 kg/m^2^) volunteered to participate. The ethics committee of the University of Potsdam approved the study (application number: 19/2018).

### Whole-Body EMS

The wireless WB-EMS device of Easy Motion Skin® (EasyMotionSkin GmbH, Leipzig, Germany) with a low-frequency neuromuscular electrical stimulation with interval stimulation pattern (pulse width 350 μs, current interval 9 s 85 Hz, and pause interval 1 s 7 Hz) was used. For WM-EMS, the participants had to wear a trunk vest, a waist belt, and cuffs on/over the thighs and upper arms for the stimulation of the eight muscle groups (upper arm, chest, shoulder, upper back, lower back, abdominal, gluteal, and upper legs). Due to the different electrical conductivity of the skin and tissues, WB-EMS intensity was adjusted individually and separately for each muscle group and the overall intensity during the WB-EMS test situations until the individual tolerated maximum amperage was reached. In contrast to previous studies that recommend a WB-EMS familiarization to achieve higher currents by the participants (Kemmler et al., 2016a, 2018; Teschler and Mooren, 2019), in this study, due to the question of the acute influence of EMS, a WB-EMS familiarization was not used.

### Test Protocol and Measurement

**Figure 1:**
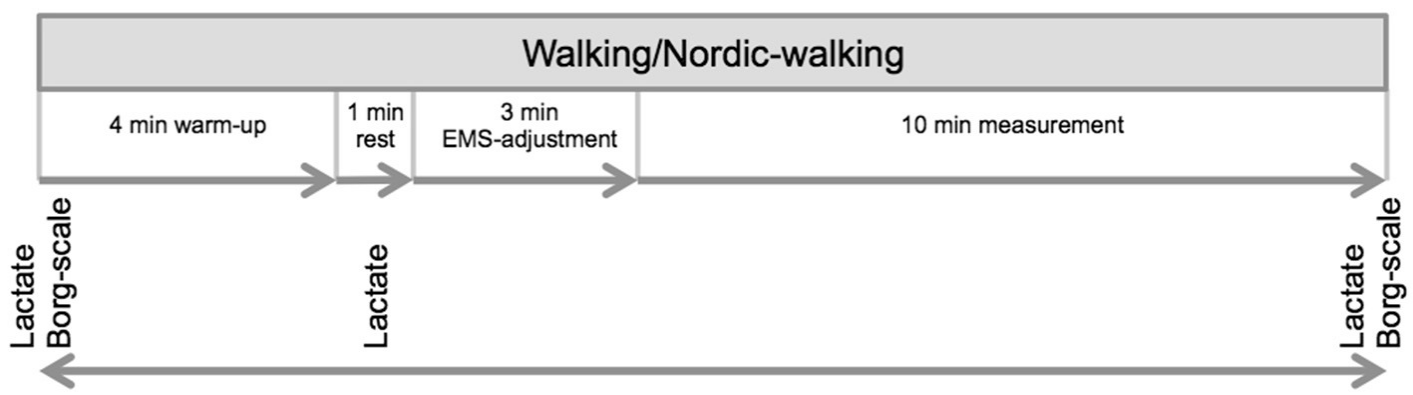
Study design. The study consisted of overall four appointments, one appointment for medical examination and first measurement and three more measurement appointments. An interval of 1 week between each measurement was implemented. During the four measurements, each condition (conventional walking, electrical muscle stimulation (EMS)-supported W, conventional Nordic walking, and EMS-supported Nordic walking) was performed in random order.

At the beginning of each measurement, participants were asked for their subjective ability to perform the test. Current body weight was measured for the calculation of relative VO_2_ (VO_2_ in relation to BW). Depending on the test situation, the participant was prepared with the WB-EMS equipment and if applicable NW sticks, which were adjusted in relation to body height (height sticks = body height × 0.68). Afterward, the treadmill test (hp cosmos® saturn 250/100 r, incline 0.4%) took place (see Figure 1 for measurement protocol). After a 4 min warm-up at a walking velocity of 5 km/h, a 1-min break took place. Afterward, the 3-min WB-EMS-adjusting period at a test velocity of 6.5 km/h and the 10 min walking test at constant velocity were performed. During the WB-EMS adjusting, stimulation intensities for the specific muscle groups and the whole body were adjusted and increased until maximum tolerated amperage of the participant. WB-EMS set intensities were noted as a dimensionless quantity (given by the system).

### Outcome Measures

Outcome measures of interest were VO_2_, relative VO_2_, blood lactate concentration, and the rate of perceived exhaustion (RPE). For assessment of the respiratory values, the wireless portable breath-by-breath system MetaMax 3B by Cortex was used (Cortex Biophysik GmbH, Walter-Köhn-Straße 2d, 04356 Leipzig/Germany, measurement accuracy ± 2.2%). The MetaMax 3B system measures volume using a bidirectional digital turbine. Using the analysis software Metasoft version 3.9.5, the respiratory data, inspiration and expiration volume, and concentration of O_2_ and CO_2_ were calculated. The analyzing system was calibrated concerning volume, pressure, and gas (calibration gas: 15% O_2_ and 5% CO_2_) before every test. An air-conditioning system guaranteed stable conditions during the whole test in the lab (STPD). VO_2_ and relative VO_2_ were reported as an average value over the 10 min test.

Capillary blood samples were taken from the earlobe before the test, in the 1 min break after warm-up and after finishing the test. Blood lactate concentration was determined with in-house used analysis devices (Biosen S-line, EKF Diagnostic Sales, Magdeburg, Germany; enzymatic-amperometric method). Subjective perception of participants (RPE) of exercise intensity was assessed using the Borg scale (6–20) before and after the test (Scherr et al., 2012).

The reliability for VO_2_peak- and RPE measurements during WB-EMS-W were determined within earlier in-house testing using the 10 m incremental shuttle walk test. Results revealed good reliability for VO_2_ peak measurements (ICC = 0.886) with a test-retest variability within the range of biological day-to-day variability for VO_2_ measurements (± 12%) and good reliability of RPE measurements (ICC = 0.859).

### Statistical Analysis

Results are presented descriptively (mean ± SD). The Kolmogorov–Smirnov test was used to test data for normality. Differences in outcome parameters between WB-EMS-W and CON-W to NW were presented relatively. BW differences between the four tests were analyzed using a one-factorial ANOVA test for repeated measures. To identify differences between WB-EMS-supported and CON-W respectively NW, two-factorial ANOVA with the single factors “EMS application” (EMS) and “(W/NW)” and its combination (EMS^*^W/NW) were performed. Paired *t*-test was used to identify differences in WB-EMS current intensities to the individual WB-EMS current intensities of the participants as well as in the mean current intensity of the eight muscle groups between the two measurement conditions. For all tests, the statistical software SPSS 23.0 was used, a significance level of α = 0.05 was assumed.

## Results

**Figure 2:**
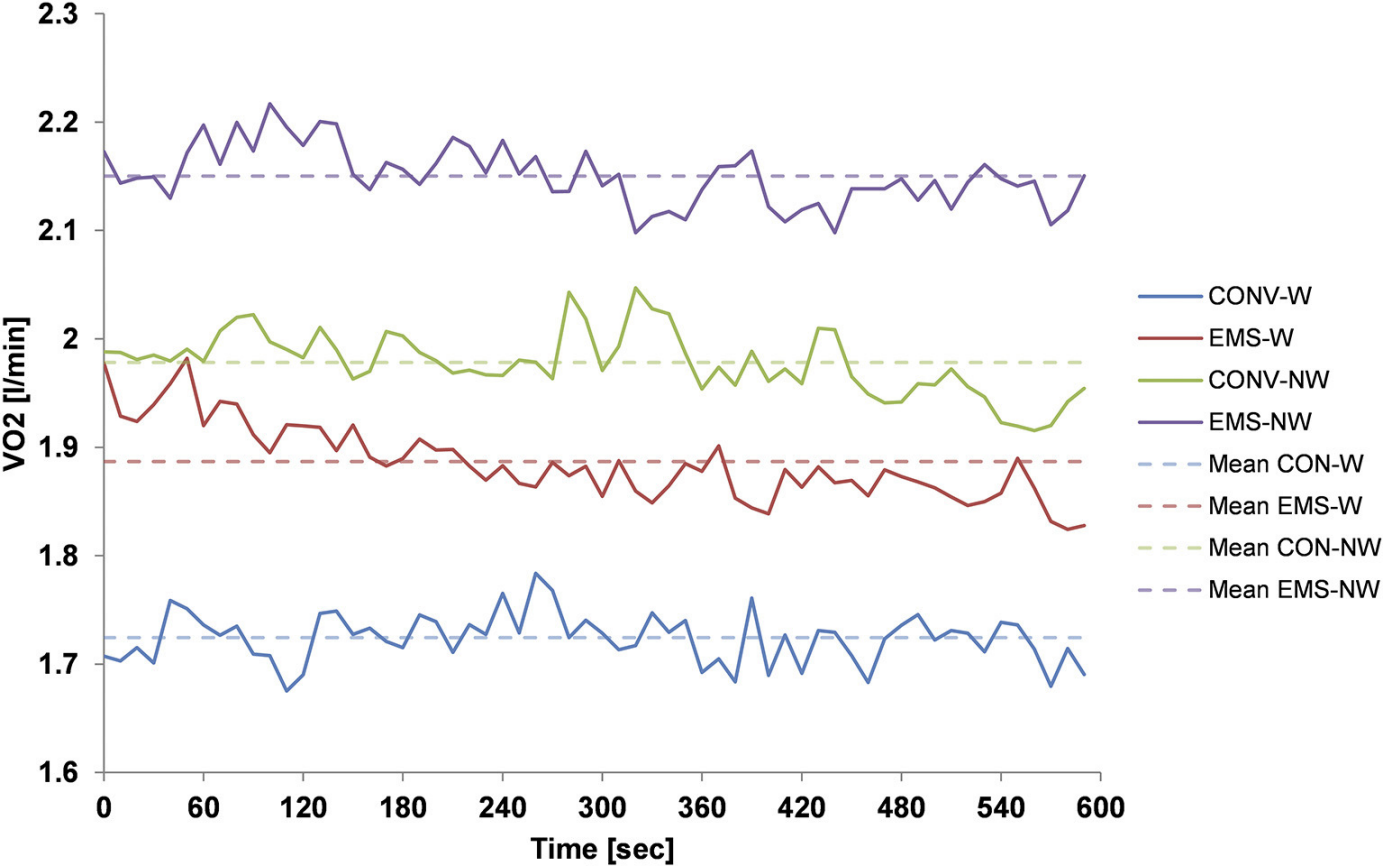
VO_2_ uptake during a 10 min treadmill test for the four different test situations: blue line indicates conventional walking (CON-W); red line indicates electrical muscle stimulation (EMS)-superimposed walking (EMS-W); green line indicates conventional Nordic walking (CON-NW); and the purple line indicates EMS-superimposed Nordic walking (EMS-NW). The solid line represents the mean on 10 s intervals for all subjects and the dashed line represents the mean value for all subjects over the 10 min measurement time. Solid shows continue steady progress in VO_2_ uptake for all measurement conditions with overlaps between two measurement conditions indicating differences in VO_2_ uptake between them. Differences in dashed lines support these results.

All subjects completed the measurements successfully and were included for data analyses. There were no dropouts and no missing data. The results of body weight, the four outcome measurements, and results of the statistical analyses are presented in Table 1. Repeated measures ANOVA test showed a *p*-value of 0.194 and revealed no statistically significant difference in BW between the four test situations. Results for VO_2_ ranged from 1.72 ± 0.15 ml/min to 2.15 ± 0.21 ml/min with *p*-values of 0.006 (EMS), 0.000 (W/NW), and 0.935 (EMS^*^W/NW). The temporal course of VO_2_ for the four test conditions is given in Figure 2. Similar results can be seen for relative VO_2_ with mean values from 20.11 ± 1.05 to 25.18 ± 1.83 ml/min/kg and *p*-values of *p* < 0.001 (EMS), *p* < 0.000 (W/NW), and 0.935 (EMS^*^W/NW).

**Table 1:** Result for the body weight, absolute (VO_2_) and relative VO_2_ (rel.VO_2_; in relation to body weight) oxygen intake, rate of perceived exhaustion (RPE; Borg-scale), and post-exercise blood lactate concentration of the outcome variables.

				**CON-W**	**EMS-W**	**CON-NW**	**EMS-NW**
VO_2_ (l/min)	Mean ± SD			1.72 ± 0.15	1.89 ± 0.14	1.98 ± 0.22	2.15 ± 0.21
	Confidence interval			(1.608–1.841)	(1.770–2.003)	(1.862–2.095)	(2.034–2.267)
	Rel. Diff. [%]			9.41		8.69	
	*p*-value^a^ (effect size)	EMS		0.006 (0.736)			
		W/NW		0.000 (0.870)			
		EMS*W/NW		0.935			
Rel.VO_2_ (ml/min/kg)	Mean ± SD			20.11 ± 1.05	22.07 ± 1.17	23.27 ± 1.74	25.18 ± 1.83
	Confidence interval			(19.16–21.06)	(21.12–23.02)	(22.32–2.095)	(24.23–26.13)
	Rel. Diff. [%]			9.77		8.20	
	*p*-value*^a^* (effect size)	EMS		0.000 (0.850)			
		W/NW		0.000 (0.937)			
		EMS*W/NW		0.952			
RPE post	Mean ± SD			11.09 ± 1.14	12.27 ± 1.60	11.64 ± 1.43	12.64 ± 1.82
	Confidence interval			(10.08–12.10)	(11.26–13.28)	(10.63–12.65)	(11.63–13.65)
	*p*-value^a^ (effect size)	EMS		0.035 (0.613)			
		W/NW		0.370			
		EMS*W/NW		0.857			
Lactate post (mmol/l)	Mean ± SD			1.14 ± 0.47	1.50 ± 0.46	1.49 ± 0.61	2.39 ± 0.88
	Confidence interval			(0.74–1.54)	(1.10–1.90)	(1.09–1.89)	(1.99–2.79)
	*p*-value^a^ (effect size)	EMS		0.003 (0.771)			
		W/NW		0.003 (0.764)			
		EMS*W/NW		0.177			
Body weight (kg)	Mean ± SD			85.9 ± 7.4	85.7 ± 6.8	85.0 ± 7.0	85.7 ± 7.2
	*p*-value^b^			0.194			

*Results are given as mean and SD and p-value for*

a *two-factorial ANOVA*.

b *ANOVA for repeated measures*. *CON-W, conventional walking; EMS-W, electrical muscle stimulation superimposed walking; CON-NW, conventional Nordic-walking; EMS-NW, electrical muscle stimulation superimposed Nordic-walking; EMS, factor electrical muscle stimulation; W/NW factor walking vs. Nordic walking*.

Post-exercise RPE ranged from 11.09 to 12.64 with a statistically significant difference for EMS as an influencing factor (*p* = 0.035). However, W/NW (*p* = 0.370) and the factor combination EMS^*^W/NW (*p* = 8.57) revealed no significant difference. The results for blood lactate concentration are in line with the results for VO_2_ and relative VO_2_ with a range of the mean values from 1.14 to 2.39 mmol^−1^, statistically significant results of the 2-factorial ANOVA for EMS (*p* = 0.003) and W/NW (*p* = 0.003), and a statistically non-significant result for EMS^*^W/NW (*p* = 0.117).

Results of WB-EMS current intensity analysis are given in Table 2. Individual WB-EMS current intensity of participants ranged from 19.9 to 29.8 with a mean ± SD of 24.7 ± 3.4 for W and from 19.5 to 32.8 with a mean ± SD of 27.7 ± 3.8 for NW. The *t*-test revealed no significant difference (*p* = 0.100). Muscle group WB-EMS current intensity ranged from 15.6 to 43.7 with a mean ± SD of 24.7 ± 8.4 for W and from 15.6 to 46.6 with a mean ± SD of 25.7 ± 3.8 for NW. The *t*-test revealed no significant difference (*p* = 0.054).

**Table 2:** Whole-body electrical muscle stimulation (WB-EMS) intensity.

**A**			**B**		
	**W**	**NW**		**W**	**NW**
01	22.4	20.6	Chest	15.6 ± 4.1	15.6 ± 3.0
02	22.6	29.0	Upper arm	18.5 ± 2.4	20.5 ± 2.9
03	21.5	23.9	Abdominal	22.2 ± 4.9	23.5 ± 5.9
04	29.8	29.6	Upper legs	43.7 ± 5.3	46.6 ± 6.5
05	26.4	25.5	shoulder	19.5 ± 5.3	21.8 ± 5.6
06	27.3	27.3	Upper back	28.7 ± 6.5	28.5 ± 6.2
07	29.8	32.8	lower back	20.2 ± 7.7	20.7 ± 6.4
08	19.9	19.5	Gluteal region	28.9 ± 5.2	28.4 ± 5.4
09	20.8	25.3	Mean ± SD	24.7 ± 8.4	25.7 ± 8.8
10	24.8	27.0			
11	22.4	22.4			
Mean ± SD	24.7 ± 3.4	27.7 ± 3.8			

*Values are given as dimensionless units by the system. (A) Individual current intensity of mean participants for both conditions (walking, W and Nordic walking, NW) as well as conditions mean and SD. Paired t-test revealed no statistical difference between the two conditions (p = 0.100). (B) Mean ± SD of current intensity for the eight muscles/muscle groups of all participants for both conditions, W and NW. Paired t-test revealed no statistical difference between the two conditions (p = 0.054)*.

## Discussion

The major finding of our study is that superimposed WB-EMS affect metabolic demand during W and NW significantly. VO_2_ uptake was higher in both EMS-W/NW when compared with CON exercises. In addition, WB-EMS leads to higher blood lactate concentrations when applied to exercise. However, clinical relevance for VO_2_ uptake and blood lactate concentration is debatable. The second finding of our study is that WB-EMS-superimposed exercises are perceived as more strenuous compared with CON exercise.

To our knowledge, this is the first investigation of a possible intensification of low-intensity exercises through superimposed WB-EMS. However, a wide range of studies already showed that EMS has the potential to increase the metabolic demands of different types of exercise, whether limited to a single muscle or related to the whole body (Buuren et al., 2015; Mathes et al., 2017). Measuring oxygen uptake relative to body weight during exercise represents the gold standard for measurements of exercise intensity. However, different outcome measures are useful to define metabolic demands and metabolic stress to identify the intensification potential of WB-EMS respectively EMS isolated for specific muscles.

As a prerequisite for evaluating the relative oxygen uptake, we analyzed the body weight difference of the participants between the four measurement appointments. ANOVA for repeated measures resulted in a *p*-value of 0.94 being synonymous with a statistically non-significant body weight difference between the four measurement appointments. Therefore, we can conclude that reported differences in relative VO_2_ are founded by differences in VO_2_ and not by variations in body weight. Amaro-Gahete et al. (2018) investigated performance-related parameters in runners and concluded that 6 weeks of WB-EMS training, coupled with a reduction in running endurance training, improved relativeVO_2max_. While VO_2_ did not improve after the intervention period, body weight reduction during that time was statistically significant. Therefore, the conclusion of a relativeVO_2max_ improvement through WB-EMS training implementation compared with the control group training conventionally with non-significant differences in VO_2_ and body weight is worth challenging. However, due to our body weight data, we can conclude the effect of WB-EMS-W/NW on relative VO_2_.

Regarding WB-EMS-superimposed cycling, several studies support our findings regarding exercise intensification through WB-EMS. Wahl et al. (2012) investigated the physiological responses and perceived exertion during an incremental cycle ergometer test. Compared to our study, EMS was just applied to the thigh and calf muscles. However, they found higher metabolic stress at 75%, respectively, 100% of peak power output when superimposed EMS was applied. In a further study, they found similar results during a 60 min constant workload test on the cycle ergometer (Wahl et al., 2014). Mathes et al. (2017) investigated the chronic effects of superimposed WB-EMS during ergometer cycling. Participants in the WB-EMS-superimposed cycling group performed the training sessions during the 4 week training intervention with a significantly higher VO_2_peak (7% higher) compared with the cycling group. Even though participants performed more demanding exercises for the metabolic and respiratory system with applied WB-EMS, a 4 week training intervention of WB-EMS-superimposed cycling exercise did not result in superior improvements of endurance capacity than cycling alone did. This fact is in line with our results that the increase of 9.41% (VO_2_) compared to 9.77% (relative VO_2_) for WB-EMS-W and 8.69% (VO_2_) compared to 8.2% (relative VO_2_) for WB-EMS-NW is within the range of biological day-to-day variability. Therefore, regardless of the fact that the differences we obtained are statistically significant (WB-EMS as factor of 2-factorial ANOVA), the intensification of W and NW through WB-EMS seems to be clinically irrelevant for this population. However, the results for combined factor analyses (EMS^*^W/NW) of the 2-factorial ANOVA test indicate that WB-EMS does not have a different impact on VO_2_ between WB-EMS-W/NW. This result contradicts our hypothesis that due to the higher muscle recruitment in various body segments during NW, WB-EMS has a higher impact on NW compared to W.

This conclusion is also confirmed by the results of the EMS current intensity analysis. Various studies recommend a WB-EMS familiarization for a higher WB-EMS current intensity toleration by the participants and ultimately higher impact of the WB-EMS (Kemmler et al., 2016a, 2018; Teschler and Mooren, 2019). Since none of the study participants has had any experience with EMS so far, we did not do this familiarization to investigate the acute impact of WB-EMS. The analysis of the EMS current intensities showed no significant differences in the individual current strength of the participants or the current strength related to the eight muscle groups.

In addition to the use of superimposed WB-EMS in endurance training, it is also used as an intensification tool in strength training. Watanabe et al. (2019) investigated metabolic differences of WB-EMS, voluntary exercise, and its combination during body weight resistance training. They found an increase in relative VO_2_ of ≈23% during WB-EMS-superimposed body weight resistance training. Compared to the difference for VO_2_, we obtained during WB-EMS-W respectively NW, the VO_2_ increase during WB-EMS-superimposed resistance training seems to be clinically relevant. One reason for this difference might be differences in the focus of the participants on the exercise. During the WB-EMS resistance training, a 4 s exercise, 4 s rest intervals were used, whereas WB-EMS was just applied during continuous dynamic exercise. Therefore, it might be possible that participants were more focused on their muscle contraction during the 4 s intervals compared with the ongoing walking tasks on the treadmill, and a higher WB-EMS intensity was tolerable leading to a higher VO_2_ increase.

An interesting result of our study is the difference in VO_2_ between W and NW. We obtained a 15.12% higher VO_2_ for CON-W vs. NW, respectively, 13.76% for WB-EMS-superimposed exercise. For relative VO_2_, differences are 15.71% (CON) and 14.09% (WB-EMS superimposed). NW has been the subject of research in previous studies, for example, Schiffer et al. (2006) investigated differences in VO_2_ between W and NW. In contrast to our results, they found a VO_2_ difference between W and NW of 8% for a similar velocity of 1.8 m/s (≈ 6.48 km/h). In contrast, Church et al. (2002) found a ≈20% higher VO_2_ during a 1,600 m walking field test at self-selected walking speed. Figard-Fabre et al. (2009) investigated the impact of a 4 week learning period of the NW technique on VO_2_ intake during a 5 min treadmill test (v = 4 km/h). In line with our results, they found an initial difference of ≈12.2% in VO_2_ between NW and W and beyond an increase of ≈17.6% after the learning period. Our participants were only fundamentally familiar with the NW technique. Therefore, it could not be excluded that a familiarization of the participants with the NW technique might have enlarged the difference in VO_2_ intake between W and NW. It can only be speculated about a stronger WB-EMS impact after an NW technique-learning period. However, a longer period of NW with additional WB-EMS might increase the VO_2_ uptake and relative VO_2_ uptake.

In addition to the measurable difference in exercise intensity, the RPE is an important factor during the investigation of new training methods. Our results suggest that both WB-EMS-superimposed exercises, W and NW, are felt as more exhausting compared to the CON exercise. The application of WB-EMS on exercise seems to be a significant influencing factor regarding RPE. In contrast to this, Wahl et al. (2012) found conflicting results for EMS-superimposed cycling on an ergometer. While there were no differences in a first examinationKlicken oder tippen Sie hier, um Text einzugeben., another study came to the result that EMS-superimposed cycling was perceived as more strenuous than cycling alone (Wahl et al., 2014). Therefore, an evaluation of our results is difficult. It is different from the comparison between W and NW for both CON and EMS superimposed. In line with the previous studies, we did not find a statistically significant difference in RPE between the two exercises for both test situations. Barberan-Garcia et al. (2015) reported that NW generates a higher exercise intensity at the same RPE compared with standard W. They investigated VO_2_ uptake and RPE (using modified Borg scale) in a group of chronic obstructive pulmonary disease patients and compared the results of two 6 min walking tests, one time during a standard W test and other time during NW. Participants did not report any differences in RPE between W and NW. Figard-Fabre et al. (2011) investigated RPE between W and NW in a group of obese middle-aged women. Results confirmed our findings of similar RPE between W and NW.

Lactate measurements represent another tool for exercise intensity evaluation. As a result of our investigation, we were able to show that WB-EMS-W/NW leads to higher lactate formation in the muscles compared with CON exercise. However, it is debatable whether the extent of this increase is relevant or not. This result supports the assumption that EMS primarily excites type 2 muscle fibers, which have a higher lactate formation compared to type 1 muscle fibers. In line with our results, Wahl et al. (2012, 2014) also found that WB-EMS-superimposed cycling leads to a significantly higher lactate formation compared with cycling alone during a 60 min constant workload and an incremental test. Due to the additional non-selective recruitment pattern of primarily fast-twitch motor units, they concluded that an intensifying impact of WB-EMS on low-intensity endurance exercise could be a result of the mild eccentric work induced by the simultaneous activation of agonist and antagonist during the cycling movement. Further research seems to be necessary to investigate the impact and type of contraction modes of WB-EMS during dynamic exercises.

There are some limitations of the current study. The high variability in the age of the included participants within this sample can be stated as a limitation of the study with regard to the results, since it would be expected that there would also be a high scatter, especially with VO_2_ and relative VO_2_. The decision for this inclusion criterion was based on an assessment of the age of a manifestation of T2DM (35 years) and the occurrence of accompanying diseases (70 years), which could influence the outcome measures. However, the low SD in the examined variables suggests that age should not be regarded as an influencing factor. This could be because the selected exercise intensity for the W and NW, both conventionally and under WB-EMS, was a low-intensity exercise in a range in which the age and the associated VO_2_ capacity of the test persons have no influence.

Our results show that the use of WB-EMS to intensify exercise is limited, at least in our subject group. An influence of the WB-EMS on exercise intensity is statistically shown but is below the threshold for clinical relevance. Therefore, the high cost of acquiring the device should be considered when using WB-EMS in different patient groups, such as T2DM and assessing training success. However, habituation of the subjects with the WB-EMS and the presumed higher tolerance to the current intensity of the WB-EMS and associated higher intensification may still change this cost-benefit consideration and indicate WB-EMS-W or NW as an effective training method in different patient groups such as T2DM.

## Conclusion

Our results indicate that WB-EMS influences parameters of exercise intensity. The impact on exercise intensity and the suitability for intensifying low-intensity exercise is questionable because of the low differences in the outcome variables. Regarding our results, CON-NW seems to be a more intensive training method than WB-EMS-W. Further studies seem to be necessary to confirm our results. An application of WB-EMS with more intensive ergometer exercises, such as elliptical machine training, could be another approach for WB-EMS-superimposed training. We acknowledge the support of the Deutsche Forschungsgemeinschaft and Open Access Publishing Fund of University of Potsdam.

## Data Availability Statement

The raw data supporting the conclusions of this article will be made available by the authors, without undue reservation.

## Ethics Statement

The studies involving human participants were reviewed and approved by Ethics Committee of the University of Potsdam. The patients/participants provided their written informed consent to participate in this study.

## Author Contributions

RV and JS conceived, designed the research, and analyzed the data. RV conducted the experiments and wrote the manuscript. JS, MH, AQ, and HV revised the manuscript. All authors read and approved the manuscript.

## Conflict of Interest

The authors declare that the research was conducted in the absence of any commercial or financial relationships that could be construed as a potential conflict of interest.

## Publisher's Note

All claims expressed in this article are solely those of the authors and do not necessarily represent those of their affiliated organizations, or those of the publisher, the editors and the reviewers. Any product that may be evaluated in this article, or claim that may be made by its manufacturer, is not guaranteed or endorsed by the publisher.
